# Outpatient Telehealth Implementation in the United States during the COVID-19 Global Pandemic: A Systematic Review

**DOI:** 10.3390/medicina57050462

**Published:** 2021-05-09

**Authors:** Cristian Lieneck, Eric Weaver, Thomas Maryon

**Affiliations:** 1 School of Health Administration, Texas State University, San Marcos, TX 78666, USA; 2 Institute for Advancing Health Value, Western Governors University, Salt Lake City, UT 84107, USA; eric.weaver@wgu.edu; 3 The School of Community and Rural Health, The University of Texas Health Science Center at Tyler, Tyler, TX 75708, USA; thomas.maryon@uthct.edu

**Keywords:** ambulatory care, outpatient care, telehealth, COVID-19, pandemic, implementation

## Abstract

*Background and objectives:* Ambulatory (outpatient) health care organizations continue to respond to the COVID-19 global pandemic using an array of initiatives to provide a continuity of care and related patient outcomes. Telehealth has quickly become an advantageous tool in assisting outpatient providers in this challenge, which has also come with an adaptation of U.S. government policy, procedures, and, as a result, organizational protocols surrounding the delivery of telehealth care. *Materials and methods:* This systematic review identified three primary facilitators to the implementation and establishment of telehealth services for the outpatient segment of the United States health care industry: patient engagement, operational workflow and organizational readiness, and regulatory changes surrounding reimbursement parity for telehealth care. *Results:* Researchers identified three barriers impacting the implementation and use of telehealth resources: patient telehealth limitations, lack of clinical care telehealth guidelines, and training, technology, and financial considerations. *Conclusions:* This systematic review’s identified facilitators and barriers for telehealth implementation initiatives in the United States can assist future outpatient providers as the global pandemic and associated public health initiatives such as physical distancing continue.

## 1. Introduction

### 1.1. Rationale

The COVID-19 global pandemic [1] continues to influence unprecedented change for the United States health care industry. Ambulatory (outpatient) care organizations continue to adjust process and service-delivery modifications in an attempt to pursue both continuity and related patient outcomes [2,3,4]. The implementation of telehealth [5] resources and extension of prior organizational telehealth initiatives assist with physical distancing requirements set by state governments and public health professionals in the United States, while also helping to achieve quality patient care and related outcomes.

The implementation and infusion of telehealth resources such as live patient–provider video conferencing sessions, audio-only teleconferences that entail multiple patient providers, among other initiatives, continue to be adapted by outpatient organizations based upon experiences and identified best practices as the pandemic lingers. To date, limited research has been recognized that focuses on this industry segment’s telehealth initiatives specific to ambulatory care organizations in the United States. Furthermore, the vast array of services (specialties) provided by the ambulatory care segment of the industry in the United States does not permit an industry-wide implementation best-practice standard due to the uniqueness of outpatient specialty and sub-specialty service delivery. This review differs from prior studies surrounding telehealth implementation during the COVID-19 pandemic by focusing specifically on outpatient (ambulatory care) health care organizations located within the United States.

### 1.2. Objectives

The objective of this systematic review is to determine the underlying themes (constructs) regarding facilitators and barriers experienced by ambulatory care organizations when implementing telehealth during the pandemic. Current best practices and other outpatient organization experiences are important to review and identify for the ongoing adaptation of processes and policies to ensure continuity of care in these facilities. However, ongoing assessment of the U.S. health care industry’s implementation of telehealth in response to the pandemic continues to demonstrate varying opinions and organization-specific success (and non-success) at the outpatient level. The research team’s focus was to evaluate such experiences in the literature, code/classify both facilitators and barriers to telehealth implementation (often initiated by organizations in a rapid manner), and to disseminate research findings to further assist the ambulatory care environment during the pandemic response.

## 2. Materials and Methods

### 2.1. Eligibility Criteria

Studies were included in this review if they focused specifically on outpatient/ambulatory care health care organizations and implemented or further adopted telehealth initiatives during the COVID-19 global pandemic. The articles had to be published in quality peer-reviewed journals. While many studies included an evaluation of patient outcomes as a result of telehealth during the pandemic, this was not a required criterion to be included in the review. An assessment of strength of evidence was conducted for each article using the Johns Hopkins evidence-based practice rating scale (JHNEBP) and only articles focused on outpatient organizations in the United States with a publication date between 1 January 2020 through 1 October 2020 were included in the review.

### 2.2. Information Sources

Three databases were queried to identify the review articles: Cumulative Index to Nursing and Allied Health Literature (CINAHL), Academic Source Complete, and PubMed (which queries MEDLINE). The database search was conducted from September 28 through 1 October 2020.

### 2.3. Search

In an effort to identify studies specific only to non-hospital, ambulatory health care organizations, an aggressive search string with Boolean operators was identified by the researchers that produced the highest initial database results. Medical Subject Headings (MeSH) is the National Library of Medicine-controlled vocabulary thesaurus utilized to index research articles for PubMed (MEDLINE) and was used to identify key words in the search. Multiple iterations of searches were conducted using various Boolean operators for all review variables in an attempt to identify the highest review sample. The final search string identified by the researchers was: “ambulatory care” OR “outpatient care” OR “outpatient services” OR “urgent care” OR “clinic visits” AND “COVID-19” OR “coronavirus” OR “2019-ncov” AND “telemedicine” OR “telehealth” OR “telecare” OR “telecommunication” OR “online” OR “virtual” providing the maximum search results.

In addition to the aggressive, recent article publication date range to specifically identify ambulatory care organizations in the United States utilizing telehealth initiatives during the pandemic, additional search criteria included English-only academic/peer-reviewed journals only, and U.S. geographic location only. While the research team acknowledges the importance of a global perspective on the implementation of telehealth during the pandemic, this review initiative was intended to specifically investigate inherent, U.S.-specific characteristics of outpatient organizational initiatives. Inherent in this investigation was to expand upon related research initiatives surrounding telehealth and the COVID-19 global pandemic and use of telehealth [6,7].

### 2.4. Initial Study Selection

This review was guided by the preferred reporting items for systematic reviews and meta-analysis (PRISMA). Three of the four researchers participated in the initial database search and any/all identified articles identified in the initial search were included in this study, regardless if a full-text version of any article was available. By not utilizing the ‘full-text only’ search criteria in the initial database search, a maximum number of initial articles was identified by the research team. Utilizing a reference management software program and capitalizing on each research team members’ home institution (university) research database access privileges, the full text of all identified articles was able to be accessed using a collective process.

Multiple research team meetings were conducted in an effort to identify any/all articles in the initial search that specifically met the study criteria. Three of the four team members participated in the initial full-text article screening and utilized a MS Excel spreadsheet to categorize and rate each article with regard to each inclusion criterion. Multiple methods were utilized in this initial review, including abstract screening, full-text article review, and also review of the initial sample articles’ literature cited/reference sections. The three reviewers providing these independent recommendations possess significant experience in outpatient/ambulatory care organization administration and collectively agreed to a final set of articles to be further analyzed. Two disagreements resulted from the initial review and the tie was broken by the third researcher participating in this stage of the review.

## 3. Results

### 3.1. Study Selection/Exclusion

**Figure 1:**
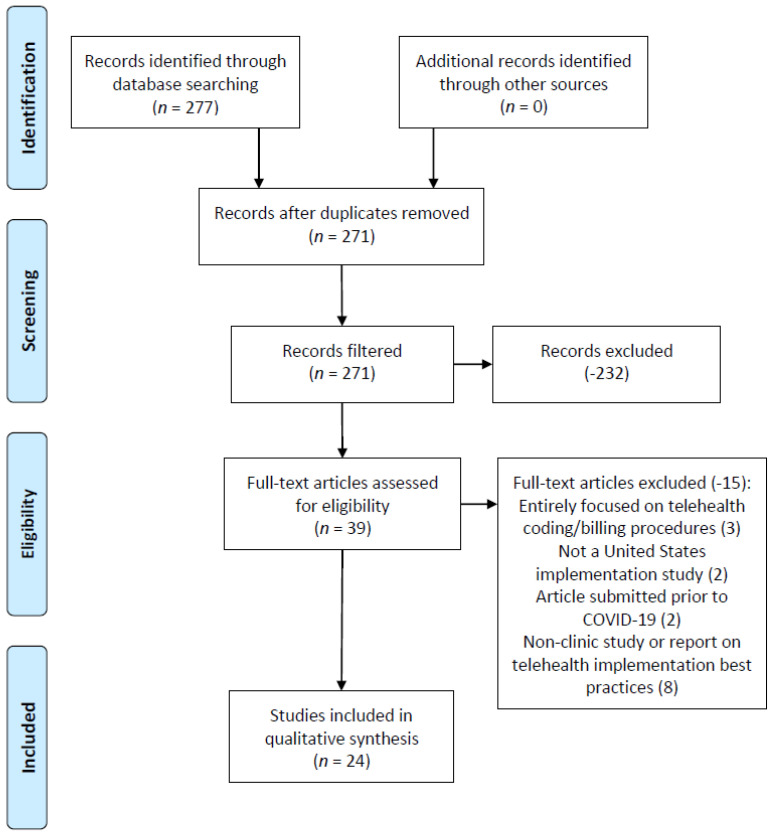
Preferred reporting items for systematic reviews and meta-analysis (PRISMA) figure that demonstrates the study selection process.

Figure 1 demonstrates the study selection and follow-on exclusion process, initially identifying 277 articles from all three research databases. Six duplicates were identified and removed across the entire search, and this study’s filtering process removed 232 articles from the initial research database query.

In addition to removing 232 articles for not meeting the study criteria, the full-text review of the remaining articles resulted in an additional 15 articles being excluded from the review. These articles were removed for the following reasons:

(a) A primary focus of telehealth coding and billing processes/procedures (3 articles),
(b) Ambulatory care organizations not located within the United States (2 articles),
(c) Articles published during the review criteria publication date range but with research conducted prior to the pandemic (2 articles), and
(d) A non-clinic or other non-health care organization-focused article (professional association and/or other non-health care delivery organization publication) (8 articles).

The eight articles omitted from the review were not directly conducted and/or focused on any specific ambulatory care/outpatient health care organization; these professional association and other related publications focusing on telehealth initiatives associated with ambulatory care during the global pandemic were reviewed by all members of the research team and all seven publications’ literature review sections were analyzed for additional articles. This action did not result in any additional articles being included in the review beyond those articles previously identified by the research team. Upon completion of the review, a total of 24 articles were included in the review.

### 3.2. Study Characteristics

Reviews entailed a systematic approach in identifying underlying characteristics associated with telehealth initiatives utilized to date during the global pandemic, specific to U.S. outpatient/ambulatory care organizations. In addition to the JHNEBP study design analysis, both facilitators and barriers with regard to the increased use of telehealth initiatives during the pandemic were summarized in Table 1. Articles are listed in alphabetical order by the first author’s last name.

### 3.3. Risk of Bias

The JHNEBP quality indicator frequencies from the sample are shown in Table 2. While it is preferred that research articles with strength of evidence ratings of level I and/or II are utilized in any systematic review, the researchers immediately identified a lack of published research in this segment of the U.S. health care industry to date. As a result, all JHNEPB strength of evidence classifications were included in this study—including level V, granted they were not letters to a journal editor, yet they were focused on initiatives related to telehealth implementation that significantly contributed to the subject of this review. The majority of articles in the sample were classified as JHNEBP level IV (70%) due to their findings and evidence that focus primarily upon the authors’ individual experiences of telehealth implementation at their own outpatient organization during the pandemic.

Inclusion of level V publications (13% of the entire sample) was permitted in this review because the researchers quickly identified that while a single industry expert authored the publication (versus multiple industry experts and/or consensus panels), these individual authors were serving as organizational representatives. Therefore, individual author efforts to convey their outpatient organization’s overall telehealth initiatives as expert opinions during the early stages of the pandemic was determined by the research team to contribute valuable, additional information to this review and to assist with future industry telehealth implementation efforts.

Moreover, this was a convenience sample taken from articles focused on the U.S. only to provide an early identification of telehealth facilitators/barriers identified in the near-term of the global pandemic. Assessment of congruence across other non-U.S. health care industries was not conducted in this research and limits the external validity of the results to an extent. This search parameter was applied to address telehealth implementation facilitators and barriers specific to the United States due to the uniqueness of this country’s health care system.

### 3.4. Additional Analysis

**Figure 2:**
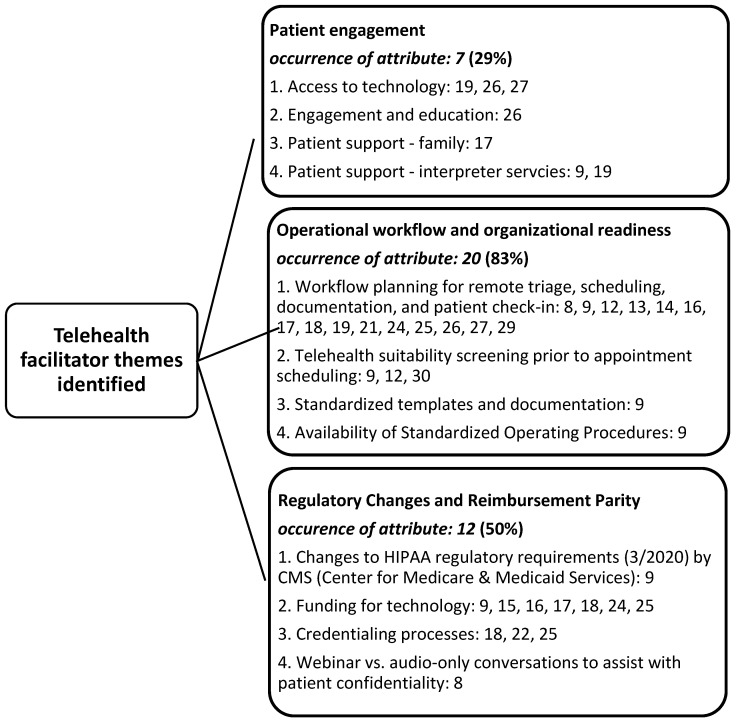
Identified themes (constructs) identified as facilitators to the implementation of telehealth during the COVID-19 pandemic in the United States.

**Figure 3:**
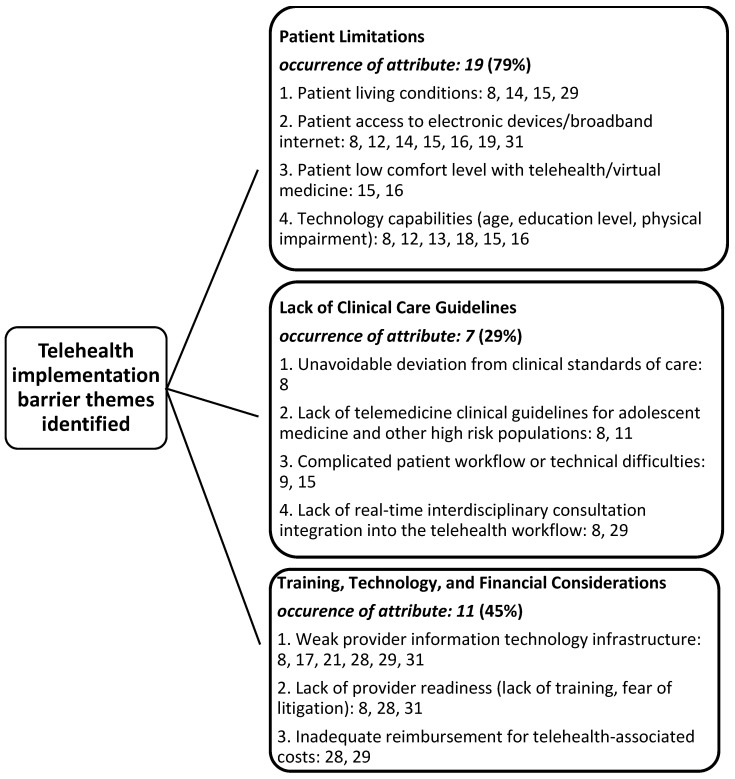
Identified themes (constructs) identified as barriers to the implementation of telehealth during the COVID-19 pandemic in the United States.

Results of the research team’s consensus meetings demonstrate three facilitator themes identified in the literature to support the adoption of telehealth resources the ambulatory care segment of the industry during the pandemic (Figure 2). Additionally, three barrier themes were also identified. These are listed in Figure 3. Findings are not mutually exclusive to only a facilitator or a barrier theme, as several articles demonstrated both constructs upon review.

## 4. Discussion

### 4.1. Summary of Evidence

The pandemic spurred a rapid rise in telehealth implementation in the United States. It has been shown in the literature that prior to the COVID pandemic, telehealth delivery has effectively reduced geographic and physical barriers to care for a variety of care modalities. The COVID pandemic has forced providers to push through historically real and perceived obstacles to achieve rapid telehealth implementation. Several facilitators and barriers have been identified that should be acknowledged to improve further implementation and to refine existing telehealth delivery approaches.

The research team identified three primary themes (constructs) associated with both facilitators and barriers influencing the implementation of telehealth in the United States during the pandemic. Facilitator variables identified were: patient engagement (29%), operational workflow and organizational readiness, and regulatory changes (83%) and reimbursement parity (50%). Likewise, identified barriers to telehealth implementation and percentage of attribute occurrence include patient limitations (79%), lack of clinical care telehealth guidelines (29%), and training/technology, and other financial considerations (45%). Within-construct sub-variables regarding facilitators and barriers for telehealth implementation were also able to be identified by the research team, identified by the article (reference) numbers in both Figure 2 and Figure 3.

### 4.2. Facilitator of Telehealth Implementation: Patient Engagement

Although the rapid change to telehealth during the pandemic disrupted care delivery workflow considerably, it was generally well received by both providers and patients. The review of literature suggests that the COVID pandemic served as a flashpoint for consumer-oriented change [9,19]. The transition towards a more innovative, technology-enabled approach to delivering care was previously suppressed because of industry inertia to adopt telehealth in lieu of the more profitable, in-person-based model of care. Patient engagement was a key driving force to positive uptake of telehealth; it can be ascertained from the literature that convenience was a main contributing factor to successful adoption [19,26]. The literature states there was a low rate of missed appointments for privately insured patients which suggests continued expansion of telehealth as a consumer expectation in the years to come [17,26,27]. There will, however, likely be continued challenges in patient engagement in the delivery of telehealth services to patients with Medicaid insurance (administered by all states in the U.S. for low-income individuals/families), as well as some patients on Medicare (administered by the U.S. federal government for the elderly population). These stakeholders may be unable to bridge the digital divide by having access to required technology or Internet access.

### 4.3. Facilitator of Telehealth Implementation: Operational Workflow and Organizational Readiness

Upon systematic review of the literature, it cannot be overstated the extent by which COVID-19 served as a catalyst for bringing telehealth to scale. Operational workflow and organizational readiness served as a key enabler to success [9,14,17,18,27]. Many organizations had already invested in the technology infrastructure necessary to deliver telemedicine services before the pandemic, yet the industry had never reached a critical mass with this modality of care [21,26]. Organizational readiness spanned beyond the technological profile to respond swiftly to the pandemic. The literature suggests that telehealth interventions were also enabled by a reduction in risks—both mental and physical—that COVID-19 presented to patients and staff [21]. The need to reduce these risks facilitated implementation capability, with the necessary agility in worker culture, to deliver telehealth at scale. During this implementation upswing, organizations were able to successfully redesign operational workflows and reorganize patient care protocols to triage telehealth interventions in accordance to care needs [8,12,14,29]. The synthesis of key enablers for implementation creates a blueprint for rapid conversion; however, the literature makes it clear that the most important element to successful telehealth implementation is decisive action. The importance of effective leadership and communication is demonstrated as the most critical variable to organizational readiness in this regard.

### 4.4. Facilitator of Telehealth Implementation: Regulatory Changes and Reimbursement Parity

Prior to the pandemic, reimbursement for telehealth and virtual visits was limited. With just a few exceptions, the Centers for Medicare and Medicaid Services only reimbursed for telehealth visits under very specific circumstances. In March 2020, following the announcement of the COVID-19 public health emergency and 1135 Waiver [29], several important telehealth-related reimbursement changes occurred [30]. This rapid change in reimbursement corresponded with less restrictive regulatory requirements at the national and state level in order to accelerate telehealth deployment during a time of crisis [17,25]. Commercial payers followed suit, thus creating an idealized regulatory and reimbursement climate to which telehealth implementation could occur at scale [31,32]. While expansion of telehealth continues under these policy changes, it is unclear whether expansion would be sustainable should these be reversed. Nonetheless, the literature points to an effective policy response that ultimately led to the intended consequence of rapid telehealth adoption [8,9,15,24]. In conjunction with patient engagement/consumerism and organizational readiness to implement, changes in regulatory policy were certainly noted as a key facilitator for telehealth implementation.

### 4.5. Barrier to Telehealth Implementation: Patient Limitations

Patient centered barriers that impact patient engagement include family support, demographic factors such as age, socioeconomic status, payor type, and both the capability and availability of needed technology [8,19]. The human–technology interface can be impeded by patient physical limitations with digital tool use. Patients with visual, hearing, cognitive impairment or language barriers require special consideration. Access to the needed technology to facilitate the telehealth interaction as well as the capability to effectively utilize technology are essential. Lack of patient education upfront on the telehealth process to set expectations can be a barrier to success [16,31]. Patient education, if not provided, can reduce the lack of patient acceptance and buy-in of virtual modalities. Lack of privacy in the home and the ability to have confidential discussions on sensitive topics such as adolescent health sexuality is a barrier that must be addressed [18,19,31]. Lastly, it is important to facilitate patient availability of equitable access to technology regardless of income, payor, or other factors [14,16].

### 4.6. Barrier to Telehealth Implementation: Lack of Clinical Care Guidelines

Lack of available clinical guidelines for specialty care when conducing telehealth interventions is a barrier that when addressed will help alleviate provider hesitancy and litigation concerns related to the lack of care standardization [8,15,29]. Telehealth limits the ability of providers to conduct certain aspects of the physical examination as well as collecting vital signs, specimens, and other clinical data remotely. Inability to perform certain types of exams or deliver certain treatment modalities will also need further technological development [11,15]. In addition, even if technology exists, there can be provider reluctance to utilize camera technology on certain body parts.

Telehealth may cause fragmentation of interdisciplinary care teams that otherwise produced well-integrated care delivery for in-person care. Interdisciplinary care is critical to address both complex clinical interactions and social factors driving poor health outcomes. Telehealth disruption of these care processes is exacerbated by complex workflow and limited technological capabilities. In addition, hesitant provider acceptance and engagement can be due to concerns around litigation for not properly assessing or managing patient care. More reason for robust telehealth clinical guidelines for care delivery [31].

### 4.7. Barrier to Telehealth Implementation: Training, Technology, and Financial Considerations

Lack of support for telehealth engagement for both patients and providers can successfully inhibit implementation. If not mitigated, information technology (IT) infrastructure challenges such as scalability, connectivity, and access to updated technology will limit uptake and reduce adoption. Technology platforms need to maintain HIPAA (Health Insurance Portability and Accountability Act of 1996) compliance supporting patient privacy and confidentiality.

Long-standing barriers to implementation have included limited reimbursement, complex licensing requirements (particularly across state lines), and administrative hurdles for providers needing credentialing for telehealth service delivery [8,17,28,29]. Lack of successful loosening of legislative reimbursement requirements for telehealth services has historically been a significant barrier [21,31]. Even though this issue has been addressed under the COVID expansion, financial challenges remain given the unexpected increases in IT costs related to scaling, new technology, and access. Additionally, reimbursement regulatory knowledge gaps and administrative billing process challenges will continue to be barriers requiring proactive attention [8,17,28,31].

## 5. Conclusions

This systematic review identified facilitators and barriers related to the implementation of telehealth services in ambulatory care organizations located in the United States. The uniqueness of the U.S. health care industry, including being the only private system in the world, suggests intricate steps and other potential processes/protocols to establish telehealth care as a rapid response to the pandemic. While facilitators and barriers identified are directly influenced by the United States health care system, this study suggests challenges and best practices offered by U.S. outpatient organizations that may also be beneficial for other countries as well.

Patient engagement, operational workflow and organizational readiness, and regulatory changes surrounding reimbursement parity for telehealth care constructs identified in the review demonstrate an ongoing initiative to implement telehealth in the outpatient organization with demonstrative patient satisfaction and optimal outcomes. Ongoing pandemic challenges related to such implementation identified in the research entail patient telehealth limitations, lack of clinical care telehealth guidelines, and training, technology, and financial considerations. These challenges, while not specifically unique to the United States health care system, do suggest inherent inequities in the delivery of care using telehealth resources, as implementation efforts continue. Future research surrounding this study’s findings include the identification of potential telehealth usage/implementation trends during the initial months of the pandemic, as well as attempting to quantify telehealth utilization efforts in the industry (and possible failed attempts). Ambulatory care providers within and beyond the United States can benefit from these telehealth implementation facilitators and barriers as the global pandemic continues.

## Figures and Tables

**Table 1:** Summary of findings (*n* = 24).

Author(s)	Participant(s)	* JHNEBP Study Design	Facilitators Leading to an Increased Utilization of Telehealth in Ambulatory care Organizations during COVID-19	Barriers Leading to an Increased Utilization of Telehealth in Ambulatory Care Organizations during COVID-19
Barney et al. [8]	Adolescent and Young Adult Medicine Clinic at the University of California San Francisco	4	Use of technology (ex. Zoom chat and/or use of earbuds) to provide additional privacy measures Point-of-care testing can be referred to external laboratory provider Use of evidence-based guidelines of clinical scoring modalities possible with telemedicine examinations Patients can submit photos for discussion	Limited patient privacy for sensitive discussions/questions Clinician decision-making limitations for point-of-care testing and physician examinations not in person Use of video for sensitive examinations was not comfortable for most patients
Bulman et al. [9]	Interventional radiology clinic	4	Development of a standard operating procedure that involves both administrative staff and physicians Pre-screen patients beforehand to determine telehealth suitability based on patient diagnosis Provide real-time staff via phone/email to assist with any patient technical issues	Audio-only multidisciplinary visits were not as optimal as virtual meeting rooms offering an ability for providers to enter/leave the patient encounter Audio-only does not offer the use of webinar breakout rooms for selected individuals in the meeting to discuss private matters
Chavis et al. [10]	Academic general pediatrics clinic	4	The primary use of telehealth in lieu of in-person patient visits supports provider health by ensuring physical distancing Quarantined providers are able to continue treating patients via telehealth A patient triage system helps identify those patients with medical needs who must be seen in person	Unaware medical providers may decide to see patients with uncommon COVID-19 symptoms (conjunctivitis) in person versus using telehealth Symptoms of COVID-19 can mirror common complaints of patients using virtual medicine
Childs et al. [11]	Intensive outpatient, group-based psychiatric (IOP) care clinic	4	Utilization of telehealth to deliver group-based treatments in high-risk populations is possible	Initiating a internal restriction on IOP referrals to those within the hospital’s own psychiatric inpatient units and emergency departments prevented referring physicians to be aware of this possibility when available to all patients
Compton et al. [12]	Cystic Fibrosis Multidisciplinary Telemedicine Clinic	4	Use of a cloud-based system allows for provider collaboration and scheduling coordination A telemedicine coordinator launches the webinar and assists with logistics during the patient visit	Pre-screened patients who do not possess the technology resources for a webinar provider visit default back to in-person-only visits Even with the correct technology, patients also experienced issues with their internet performance level/bandwidth availability
Dewar et al. [13]	Geriatrics primary care clinic	4	Epic “superusers” were used to help physicians set up the application for video visits Physicians were able to self-schedule virtual visits due to a newly upgraded feature in the EHR Adaption of the physical examination was established for inspection, palpation, percussion, and auscultation	Patients were initially reluctant to install video-capable applications onto their smartphones and tablets
Eberly et al. [14]	Academic health system outpatient cardiovascular clinic	2	n/a	More seamless translation services spanning an entire virtual patient encounter, from scheduling to follow-up visit/testing, are needed Strategies to improve distribution of devices with video capability or to provide broadband internet coverage could improve access
Grossman et al. [15]	Neurology outpatient clinic	4	Devices with rear and front-facing cameras performed more optimal than single-view laptop cameras, etc. Using an assistant in the patient’s residence, etc., helps with obtaining a comprehensive history and performing virtual exams Providers more efficient when using a device for video conferencing with the patient while using another computer to access the EHR	Device screen size (for providers) can be too small for certain neurological examination and/or imaging review Some detailed neurologic examinations deemed unsuitable for virtual visits (logistic constraints)
Knudsen et al. [16]	NYC Health + Hospitals	5	Maintain an ability to remain sensitive to the barriers of language, cost, and health and technical literacy prevalent in our safety-net patient population A telehealth survey assisted with assessing patient readiness for virtual care Focus on implementation with rapid improvements rather than perfect execution	Video visits were more challenging and ultimately required in-person and virtual navigators to facilitate the service for patients Safety-net systems face chronic clinician shortages, especially during a pandemic Without payment parity between virtual and in-person care inequities to access to care exists based upon patient payer type
Knopf [17]	University of Washington, the Seattle Children’s Research Institute, and Seattle Children’s Hospital psychiatry clinics	5	Possibly more family-friendly, as well as ecological Development of a protocol to convert established patients from in-clinic to telemedicine sessions with their same clinician Phone sessions that required much less bandwidth and therefore continued to be the major platform for patient care while awaiting reliable availability of the platform	Concerns exist: privacy, security of technology platforms, management of crises including suicidality, and disclosure of information in case of emergency Processing applications for hospital privileging for telemedicine providers caused treatment delays Trouble determining how many families could have access to private and secure technology at home
Loeb et al. [18]	Orthopedic surgery department	5	Patient triage ensured successful selection of patients for telehealth use beforehand Providers to consider using a lens cover to avoid unintentionally capturing video of other patients For advanced practice providers and the addition of a virtual practice location to their state licenses was determined to be unnecessary Use of direct-to-patient marketing channels to contact patients via e-mail or text message to inform of the new functionality	Patients deemed ineligible for telehealth visits included suture or staple removal, the need for a cast change, and the need for a hands-on clinical examination to determine appropriate treatment of an acute injury Bandwidth delay occasionally interrupts the smooth flow of discussion Greatest ongoing challenge was managing cameras, microphones, and software on patients’ devices to allow HIPAA-compliant video communication
Madden et al. [19]	Prenatal medical practices	3	Development of guidelines regarding which antenatal visits are appropriate for telehealth Development of guidelines regarding frequency and interval of ultrasound monitoring	Additional office staff were required to rapidly enroll patients in Epic Additional training for office staff was required specifically to schedule and manage telehealth appointments
Mann et al. [20]	Large academic health care system with an existing telehealth infrastructure	4	An aspiration of the industry for years has materialized in a matter of days due to COVID-19 Enabled the mobilization of quarantined but asymptomatic providers, mitigating the loss of highly needed resources	n/a
Savage et al. [21]	Wound care clinic	4	Comparable accuracy of store-and-forward telemedicine photography to in-person assessment Personal protective equipment resource preservation Comparable accuracy and outcomes of telemedicine videoconferencing to in-person visits	Difficulty using photographs in the evaluation of wound drainage, edema, and depth Loss of sensation and odor in clinical assessment Need for assistance from patients’ family members or caregivers to aid in photographing, videoconferencing, or dressing wound
O’Hara et al. [22]	Pediatric weight management clinic	4	Pre-planning meetings between distant and originating site colleagues Level of trust with the originating site was achieved through frequent communication to review the virtual protocol and outline roles and responsibilities proactively	Acquiring accurate vital signs and weight are key concerns especially as these data impact medical decisions and management Access and sustainability risk due to high attrition rates Fee variance between in-person visit charges (professional fee and facility fee) and telemedicine visit charges (professional fee only)
Panzirer [23]	Virtual specialty diabetes clinic	4	Increases in glucose monitoring satisfaction, trust, hypoglycemia confidence, and diabetes technology attitudes Decreases in diabetes management distress, emotional burden, and behavioral burden	Manufacturers need to allow patients with diabetes the ability to automatically import their data from the devices into one standard report even if not the manufacturer’s software
Peahl et al. [24]	Obstetrical clinic providing prenatal care	4	Establishment of a multistakeholder team, including experts in medical care Intersperse virtual visits between the in-person visits, creating critical touchpoints for services Creation of an online program modeled on group prenatal care that provides social connection and peer mentoring	Some populations may be disadvantaged by telemedicine (rural settings, low socioeconomic status with no stable internet connection
Segal et al. [25]	Clinical pharmacy within an integrated health care system	4	Patients are eligible to receive a telehealth appointment they do not require a physical examination, narcotic medication changes, or other in-person services the same day Medical assistants or clinic support staff to help set up and room the patient in the virtual waiting room prior to the telehealth visit	Both parties must stick to their scheduled appointment time Phone visits permit the pharmacist to review records during the visit. However, in a telehealth appointment more advanced preparation is required to ensure that a provider minimizes distractions or lack of eye contact with the patient when reviewing notes
Smith et al. [26]	Multispecialty physician groups	4	Use of alternative audiovisual tools (options) if one does not currently exist in the practice’s electronic medical record Investment in at least 1 h (or more) training of physicians and staff to conduct virtual visits Patient education beforehand to manage expectations	Adequate bandwidth and a secure connection to allow for proper operation of EMR-based communications Extensive codes to document virtual care/patient visits
Tanaka et al. [27]	Orthopedic medical practice	4	Significant research conducted regarding the best practices surrounding the virtual orthopedic physician examination	Reliability of virtual orthopedic visits yet to be conducted
Varma et al. [28]	Cardiovascular medical practice	4	Use of wearables such as watches, smartphones, and smart beds (with elimination of cables and skin electrodes) for in-hospital telemetry is a novel approach for intensive monitoring extending beyond the hospital environment Pandemic experience should serve as an impetus to expedite the resolution of persistent digital validation challenges	n/a
Wood et al. [29]	Hospital-based specialty clinical program provides nonprimary care management of gender-affirming care, eating disorders, HIV, adolescent gynecology and contraception, general AM, and substance abuse disorders	3	Potential unmeasured gains in health care delivery from telehealth as well which should be measured in future studies (travel, lodging, and time costs) Given the proper resources and support, achievement of broad, rapid, Telehealth scale-up is achievable	Potential emerging disparities by race It is unclear which patients will benefit most from telehealth or in-person visits and therefore clinical decision-making tools will need to be developed and tested
Wosik et al. [30]	Multiple health care delivery settings/organizations	3	Various telehealth encounters/venues offer a variety of cited opportunities A key transformation of telehealth systems is to shift from crisis mode to sustainable, secure systems that properly preserve data security and patient privacy and that offer sustained technical support for postcrisis care	Various telehealth encounters/venues offer a variety of cited barriers A post-pandemic initiative will require the re-evaluation of regulation and policies and reimbursement models across multiple stakeholders including local health care organizations, state medical board, federal government, and payers
Yellowlees et al. [31]	Outpatient psychiatric clinic	4	Decisive action cited as contributing to successful implementation Successful communication of the plan and process to both staff, providers, and patients Provider training cited	Inability to contact all patients in the short amount of time when COVID-19 became prevalent Audio-only (phone) communication used for mostly elderly patients without video capability Telepsychiatry being conducted at providers’ homes on personal computers without pre-loaded EMR software, etc.

* Johns Hopkins Nursing Evidence-Based Practice (JHNEBP) levels of strength of evidence: Level 1, experimental study/randomized control trial (RCT); Level 2, quasi-experimental study; Level 3, non-experimental, qualitative, or meta-synthesis study; Level 4, opinion of nationally recognized experts based on research evidence/consensus panels; Level 5, opinions of industry experts not based on research evidence.

**Table 2:** Summary of quality assessments.

Strength of Evidence	Frequency
II (Quasi-experimental)	1 (4%)
III (Non-experimental, qualitative)	3 (13%)
IV (Opinion of nationally recognized experts based on research evidence/consensus panels)	17 (70%)
V (Opinions of industry experts not based on research evidence)	3 (13%)

## Data Availability

Not applicable.
